# Prognostic value of radiological findings indeterminate for UIP pattern and anterior upper lobe honeycomb-like lesion in chronic fibrosing interstitial lung disease associated with MPO-ANCA

**DOI:** 10.1186/s12890-021-01718-w

**Published:** 2021-11-03

**Authors:** Hideaki Yamakawa, Shintaro Sato, Tomohiko Nakamura, Tomotaka Nishizawa, Rie Kawabe, Tomohiro Oba, Masanobu Horikoshi, Keiichi Akasaka, Masako Amano, Kazuyoshi Kuwano, Hiroki Sasaki, Hidekazu Matsushima

**Affiliations:** 1 Department of Respiratory Medicine, Saitama Red Cross Hospital, 1-5 Shintoshin, Chuo-ku, Saitama, 330-8553 Japan; 2 Department of Respiratory Medicine, Tokyo Jikei University Hospital, 3-25-8 Nishi-shimbashi, Minato-ku, Tokyo, 105-8461 Japan; 3 Department of Rheumatology, Saitama Red Cross Hospital, 1-5 Shintoshin, Chuo-ku, Saitama, 330-8553 Japan; 4 Department of Radiology, Saitama Red Cross Hospital, 1-5 Shintoshin, Chuo-ku, Saitama, 330-8553 Japan

**Keywords:** Anterior upper lobe honeycomb-like lesion, Indeterminate for usual interstitial pneumonia, Interstitial lung disease, Microscopic polyangiitis

## Abstract

**Background:**

Myeloperoxidase antineutrophil cytoplasmic antibody (MPO-ANCA) is often positive in patients with interstitial lung disease (ILD), which is also often present in patients with microscopic polyangiitis (MPA). A possible association between MPO-ANCA, MPA, and idiopathic ILD remains unclear. The objective of this study was to determine whether high-resolution computed tomography (HRCT) classification based on recent idiopathic pulmonary fibrosis guideline and specific CT findings can obtain new knowledge of prognostic factors in all MPO-ANCA-positive patients with ILD including both idiopathic ILD and MPA-ILD.

**Methods:**

We analyzed 101 consecutive MPO-ANCA-positive patients with respiratory disease. We assessed the diagnostic accuracy of CT findings, HRCT pattern, and specific radiological signs. Prognostic predictors were determined using Cox regression models.

**Results:**

Subjects with chronic ILD included 22 patients with MPA-ILD and 39 patients with ILD but without MPA. A quarter of the patients were radiological indeterminate for usual interstitial pneumonia (UIP) pattern, which resulted in a better prognosis than that for UIP pattern. “Increased attenuation around honeycomb and traction bronchiectasis” and “anterior upper lobe honeycomb-like lesion” were found to be highly frequent radiological findings (39% and 30%, respectively). In addition, the latter finding was a significant negative prognostic factor.

**Conclusions:**

Radiological indeterminate for UIP was a useful HRCT classification in MPO-ANCA-positive patients with ILD. In addition, anterior upper lobe honeycomb-like lesion was found to be specific radiological finding that was a significant prognostic factor. The present results might aid in the assessment of appropriate strategies of diagnosis in these patients.

## Introduction

Antineutrophil cytoplasmic antibody (ANCA) is well known to be pathogenic and to have diagnostic value for ANCA-associated vasculitis, and thus, myeloperoxidase-ANCA (MPO-ANCA) has been thought to be related to the pathogenesis of microscopic polyangiitis (MPA) [1]. Although MPO-ANCA positivity is often found in patients with interstitial lung disease (ILD) in clinical practice, a possible association between MPO-ANCA, MPA, and idiopathic interstitial pneumonias (IIPs) remains unclear. Some recent studies showed that MPO-ANCA positivity was associated with subsequent MPA development in patients initially diagnosed as having IIPs, especially usual interstitial pneumonia/idiopathic pulmonary fibrosis (UIP/IPF) [2–4]. Although Katsumata et al. noted that there is no consensus on whether MPO-ANCA-positive patients with ILD but without other manifestations of systemic vasculitis should be considered to have “pulmonary limited vasculitis” as a phenotypic variant of MPA, this point remains controversial [5]. However, there are few reports on the prognostic analysis of all MPO-ANCA-positive patients with ILD including those with both IIPs and MPA. Moreover, although most reports mentioned that UIP pattern was the main pattern on high-resolution computed tomography (HRCT), an overlapping or indeterminate pattern (i.e., unclassifiable pattern other than UIP, non-specific interstitial pneumonia, and organizing pneumonia) may present to some extent in the MPO-ANCA-positive population with ILD because pathologically, MPO-ANCA-positive patients with ILD showed more prominent inflammatory cell infiltration and cellular bronchiolitis [2, 6]. To clarify these issues, we aimed to investigate prognostic factors of mortality in MPO-ANCA-positive patients with ILD by using HRCT pattern classification based on the recent IPF guideline [7].

## Materials and methods

### Study sample

**Fig. 1:**
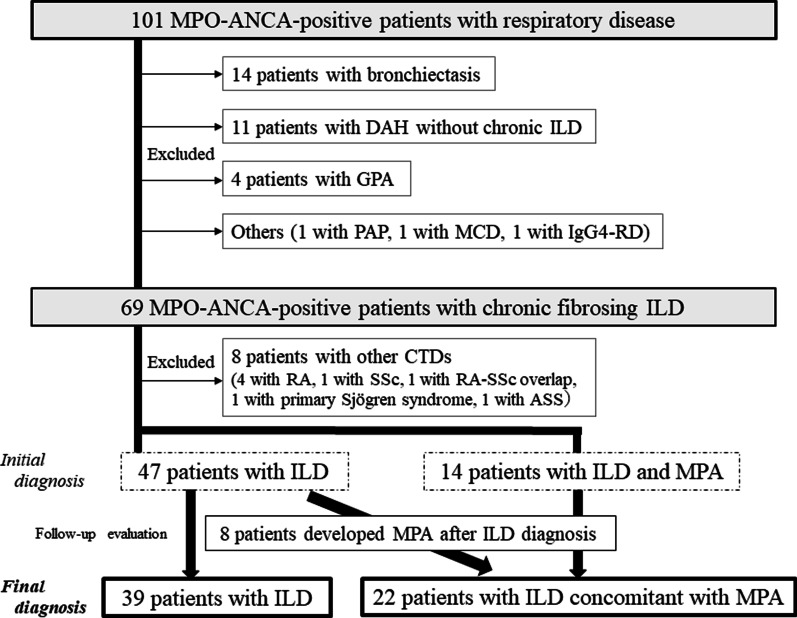
Flow diagram of the MPO-ANCA-positive patients with ILD. *CTD* connective tissue disease, *DAH* diffuse alveolar hemorrhage, *GPA* granulomatosis with polyangiitis, *IgG4-RD* immunoglobulin G4-related disease, *ILD* interstitial lung disease, *MCD* multicentric Castleman’s disease, *MPA* microscopic polyangiitis

This study was approved by the institutional review board of Saitama Red Cross Hospital (approval no. 20-U), which waived the need for patient approval or informed consent because the study involved a retrospective review of clinical records. We surveyed 101 MPO-ANCA-positive patients with respiratory disease at our hospital between January 2011 and December 2019 (Fig. 1). A definitive diagnosis of MPA, when present, was determined according to the 2017 guidelines on management of vasculitis syndrome in Japan [8], which are based on the 2012 Revised International Chapel Hill Consensus criteria [9]. Among the MPO-ANCA-positive patients with respiratory disease, 14 patients with bronchiectasis, 11 with diffuse alveolar hemorrhage (DAH) without chronic ILD, 4 with granulomatosis with polyangiitis, and 3 with a diagnosis other than MPA (i.e., pulmonary alveolar proteinosis [n = 1], multicentric Castleman disease [n = 1], and IgG4-related disease [n = 1]) were excluded. We extracted the MPO-ANCA-positive patients with chronic ILD and then collected data from each of these patient’s medical records that included characteristics, laboratory data, pulmonary function results, and chest CT findings at the time of the ILD diagnosis. We extracted the baseline clinical measurements that were obtained within 3 months of the initial diagnosis of ILD at our hospital. Survival was defined as the time from ILD diagnosis to death or date of censoring. Acute exacerbation (AE) of ILD was defined based on a previous report [10].

### Radiological analysis

**Fig. 2:**
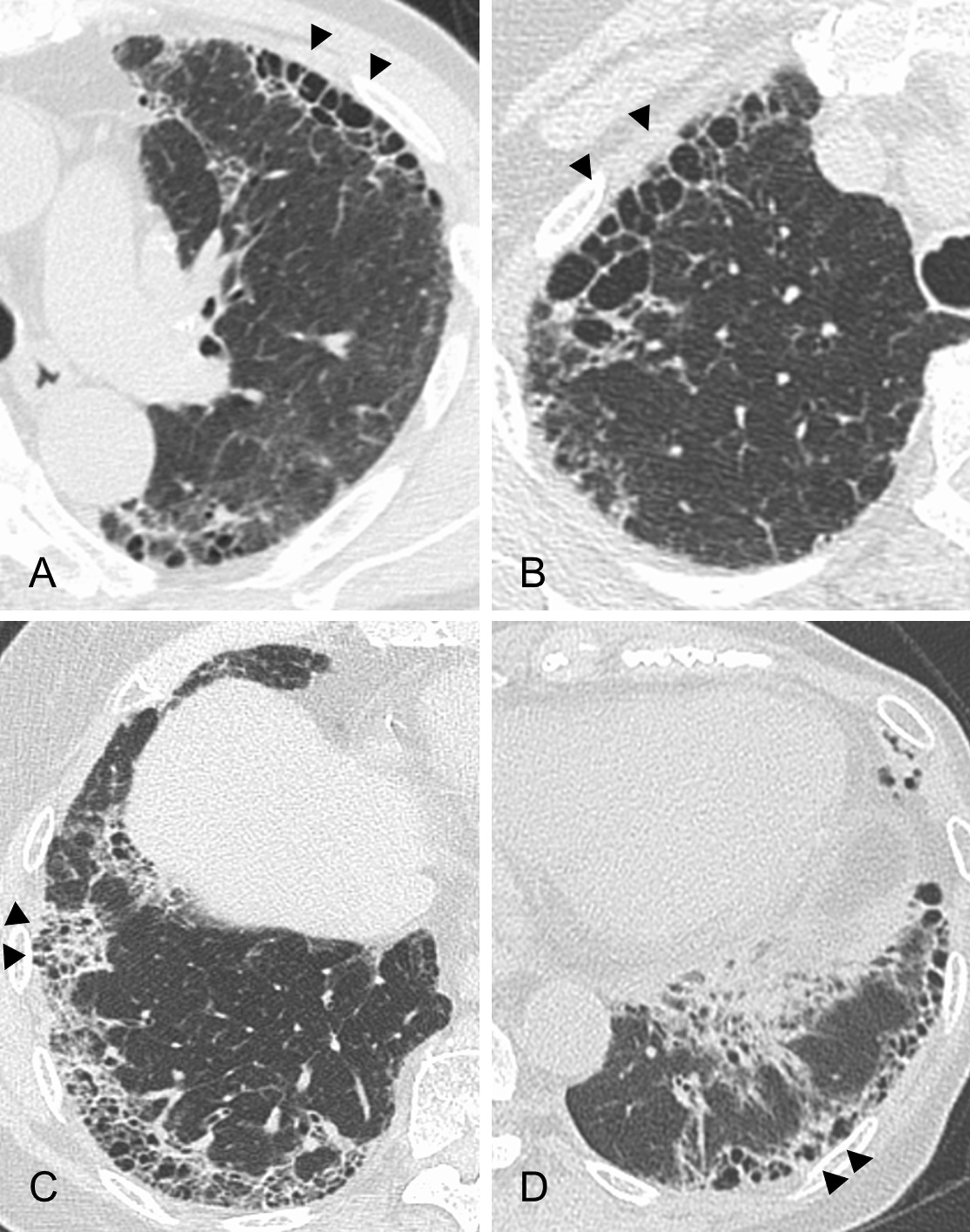
**A**, **B** High-resolution computed tomography scans of anterior upper lobe honeycomb-like lesions showing a concentration of cystic air spaces within the anterior aspect of the upper lobes (arrowheads). **A** A 69-year-old man. **B** A 72-year-old woman. **C**, **D** High-resolution computed tomography scans of increased attenuation around honeycomb or traction bronchiectasis (arrowheads). **C** A 77-year-old woman. **D** A 73-year-old woman

Each subject’s radiological findings were reviewed by two expert pulmonologists (SS and HM) who were blinded to the subject’s clinical data. Patients were classified as presenting a HRCT pattern of UIP, probable UIP, indeterminate for UIP, or alternative diagnosis according to the recent IPF guideline [7]. In the HRCT classification of this study, the presence of co-existing small airway disorders such as mosaic attenuation, centrilobular micro-nodules, and bronchial wall thickening was ignored because a high prevalence of airway abnormalities has been observed in the MPA population as being associated with rheumatoid arthritis (RA) [11, 12]. In addition, subpleural subtle reticulation (i.e., early UIP) in patients indeterminate for UIP was not included in the present study. For combined pulmonary fibrosis with emphysema (CPFE), positive findings of emphysema were visually defined as the presence of an area of low attenuation indicating the lack of a distinct alveolar wall threshold over 10% [13]. Honeycomb was defined as clustered cystic air spaces with well-defined walls and typically comparable diameters of 3–10 mm in subpleural and lower lobes [7]. “Anterior upper lobe honeycomb-like lesion” was recently reported by our group, which was defined as a modifying anterior upper lobe sign reported by Chung et al. [14, 15]. This term represents a concentration of cystic air spaces within the anterior aspect of the upper lobes (Fig. 2A, B). “Increased attenuation around honeycomb or traction bronchiectasis” was defined as an area of high attenuation around a fibrotic area such as honeycomb or traction bronchiectasis according to previous reports (Fig. 2C, D) [2, 16]. Disagreements between the two pulmonologists after the first assessment were resolved by discussion.

### Statistical methods

Categorical baseline characteristics are summarized by frequency and percentage, and continuous characteristic are reported as the mean ± SD. To detect differences between groups, Fisher’s exact test, one-way analysis of variance (ANOVA), unpaired *t*-test, Kruskal–Wallis test, or Mann–Whitney *U* test was used as appropriate. Regarding the HRCT findings, the κ value was calculated for agreement between two expert pulmonologists for the baseline assessment. We investigated potential risk factors of mortality for each variable chosen for entry into univariate Cox regression analysis and performed multivariate Cox regression analysis with the forced entry method. The Kaplan–Meier method and log-rank test were used to display and compare survival curves for the cohort stratified by each group. We considered *P* < 0.05 to indicate statistical significance. All data were analyzed with SPSS version 22.0 (IBM Japan, Tokyo, Japan).

## Results

### Overall patient characteristics

The study cohort included 69 MPO-ANCA-positive patients with chronic ILD. Among these patients, 4 patients with RA, 1 with systemic sclerosis (SSc), 1 with RA-SSc overlap, 1 with primary Sjögren syndrome, and 1 with antisynthetase syndrome were excluded. After the diagnosis of ILD at the initial visit in the patients with MPO-ANCA negativity, conversion to MPO-ANCA positive occurred in 9 patients (median follow-up: 3 years; range 1.3–7.7 years) who were included in this study (during follow-up, 2 of these patients developed MPA). In contrast, 8 patients (i.e., 17% of the subjects with MPA) developed MPA after the ILD diagnosis (median follow-up: 1.25 years; range: 0.5–6.7 years). Finally, we identified 61 patients with MPO-ANCA-positive ILD, which included 39 patients with ILD (non-MPA-ILD) and 22 patients with ILD concomitant with MPA (MPA-ILD) (Fig. 1, Table 1). The patients were observed over a median follow-up period of 3.6 years (range: 0.1–11.9 years). The MPA-ILD patients had a higher body mass index and high frequency of UIP pattern (22.7%) and anti-inflammatory agent use (95.5%) during follow-up than did the non-MPA-ILD patients. In contrast, the patients with non-MPA showed a high frequency of indeterminate for UIP pattern (35.9%). Observer agreement (*κ* value) for each HRCT classification was good at 0.767 (Table 2). The other factors (i.e., age, sex, smoking habit, frequency of each CT sign, and pulmonary function measures) were not significantly different between the MPA and non-MPA patients. Overall, the frequency of anterior upper lobe honeycomb-like lesion was 29.5% (*κ* value: 0.796), of which increased attenuation around honeycomb or traction bronchiectasis was 39.3% (*κ* value: 0.624). Among 9 patients with a history of AE of ILD during follow-up, 3 patients received an anti-inflammatory agent and only 1 patient received an anti-fibrotic agent at the time of the AE. Contrastingly, in the 6 patients who developed DAH, none of them received both an anti-inflammatory and anti-fibrotic agent at the time of DAH.

**Table 1:** Patient characteristics

	All subjects (n = 61)	MPA-ILD (n = 22)	Non-MPA-ILD (n = 39)	*P* value
Age, mean ± SD	75.1 ± 8.8	75.1 ± 9.2	75.2 ± 8.2	0.973
Male, N (%)	31 (50.8%)	9 (40.9%)	22 (56.4%)	0.293
Current or ex-smoker, N (%)	35 (57.4%)	11 (50.0%)	24 (61.5%)	0.428
BMI, kg/m^2^, mean ± SD	22.1 ± 3.1	23.2 ± 3.3	21.5 ± 2.9	**0.037**
Acute exacerbation of ILD, N (%)	9 (14.8%)	1 (4.5%)	8 (20.5%)	0.138
Diffuse pulmonary hemorrhage, N (%)	6 (9.8%)	6 (27.3%)	0 (0.0%)	** < 0.001**
KL-6 (U/mL), mean ± SD (available N = 56)	913.9 ± 824.4	726.2 ± 594.3	1010.3 ± 913.0	0.225
SP-D (ng/mL), mean ± SD (available N = 40)	151.9 ± 116.8	91.9 ± 76.8	169.3 ± 121.5	0.080
CRP (mg/dL), mean ± SD (available N = 61)	1.8 ± 2.7	1.6 ± 2.5	2.1 ± 3.0	0.475
%FVC, mean ± SD (available N = 31)	91.2 ± 19.1	98.3 ± 12.5	87.9 ± 21.0	0.160
FEV_1_/FVC ratio, % mean ± SD (available N = 31)	77.3 ± 9.4	78.8 ± 9.8	76.5 ± 9.4	0.540
%DL_CO_, mean ± SD (available N = 29)	81.5 ± 22.0	84.6 ± 15.5	79.9 ± 24.9	0.594
CPI, mean ± SD (available N = 29)	27.7 ± 18.3	24.1 ± 13.1	29.6 ± 20.5	0.445
Anti-inflammatory agent (during follow-up), N (%)	35 (57.4%)	21 (95.5%)	14 (35.9%)	** < 0.001**
Anti-fibrotic agent (during follow-up), N (%)	4 (6.6%)	0 (0.0%)	4 (10.3%)	0.287
Deaths (during follow-up), N (%)	25 (41.0%)	9 (40.9%)	16 (41.0%)	> 0.999
Median follow-up, years (range)	3.6 (0.1–11.9)	3.2 (0.1–11.9)	3.8 (0.2–11.7)	0.376

*BMI* body mass index, *CPI*  composite physiological index, *CRP* C-reactive protein, *DL*_*CO*_ diffusing capacity of the lung for carbon monoxide, *FVC* forced vital capacity, *FEV*_*1*_ forced expiratory volume in 1 s, *ILD* interstitial lung disease, *KL-6* Krebs von den Lungen-6, *MPA* microscopic polyangiitis, *SP-D* surfactant protein-D

**Table 2:** HRCT classification and signs

	*κ* value (95% CI)	MPA-ILD (n = 22)	Non-MPA-ILD (n = 39)	*P* value
No. of patients		22	39	
*HRCT pattern, N (%)*				
UIP	0.767 (0.641–0.893)	5 (22.7%)	4 (10.3%)	**0.030**
Probable UIP		13 (59.1%)	17 (43.6%)	
Indeterminate for UIP		1 (4.5%)	14 (35.9%)	
Alternative		3 (13.6%)	4 (10.3%)	
*CT signs, N (%)*				
Upper lobe-predominant fibrosis	1.000 (1.000–1.000)	0 (0.0%)	1 (2.6%)	> 0.999
Emphysema	0.756 (0.550–0.962)	1 (4.5%)	8 (20.5%)	0.138
Traction bronchiectasis	0.660 (-0.001–1.321)	21 (95.5%)	39 (100.0%)	0.361
Ground glass opacity	0.741 (0.572–0.910)	13 (59.1%)	28 (71.8%)	0.397
Consolidation	0.678 (0.431–0.924)	2 (9.1%)	5 (12.8%)	> 0.999
Mosaic attenuation (air trapping)	0.399 (0.140–0.657)	4 (18.2%)	13 (33.3%)	0.247
Anterior upper lobe honeycomb-like lesion	0.796 (0.641–0.952)	6 (27.3%)	12 (30.8%)	> 0.999
Increased attenuation around honeycomb or traction bronchiectasis	0.624 (0.430–0.817)	7 (31.8%)	17 (43.6%)	0.423
Honeycomb	0.711 (0.535–0.887)	6 (27.3%)	9 (23.1%)	0.763
Pleural thickening or effusion	0.545 (0.310–0.779)	3 (13.6%)	7 (17.9%)	0.735

*CI* confidence interval, *CT* computed tomography, *HRCT* high-resolution CT, *ILD* interstitial lung disease, *MPA* microscopic polyangiitis, *UIP* usual interstitial pneumonia

### Survival

**Fig. 3:**
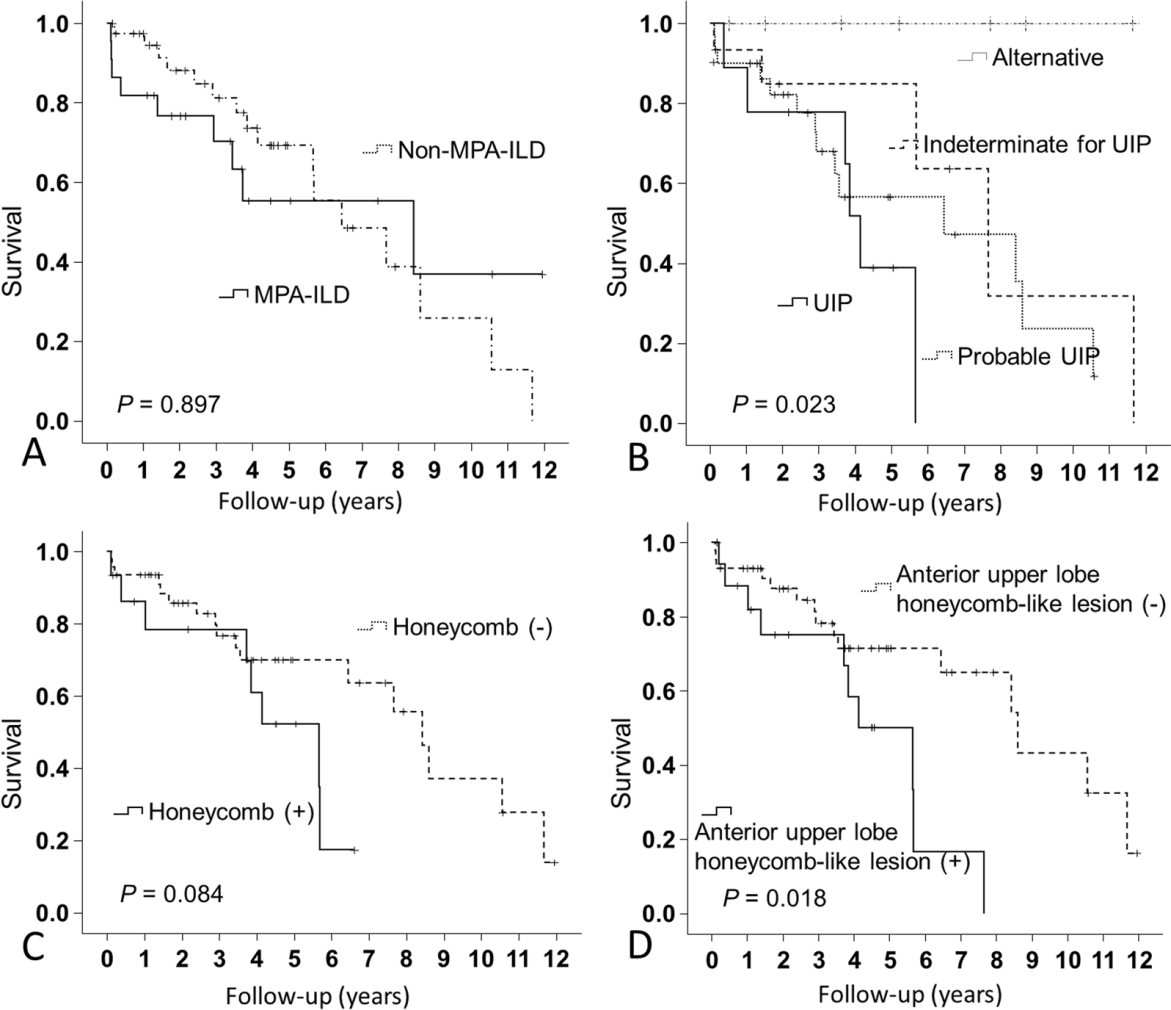
Kaplan–Meier survival curves of all-cause mortality. **A** There was no difference in survival between patients with and without MPA (*P* = 0.897). **B** Patient survival was better for those with indeterminate for UIP and alternative diagnosis than for UIP HRCT pattern (*P* = 0.023, *P* = 0.011, respectively) (overall analysis: *P* = 0.023) **C** Survival of patients with honeycomb lesions showed a worse tendency than that for patients without this finding although the difference was not significant (*P* = 0.084). **D** The patients with anterior upper lobe honeycomb-like lesions showed significantly poorer survival than those without this finding (*P* = 0.018). *HRCT* high-resolution computed tomography, *MPA* microscopic polyangiitis, *UIP* usual interstitial pneumonia

During the follow-up period, 26 patients died (43%), with 10 (45%) deaths occurring in the MPA-ILD group and 16 (41%) occurring in the non-MPA-ILD group (Table 3). Log-rank tests showed that there was no difference in survival between the patients with and without MPA-ILD (*P* = 0.897) (Fig. 3A). The respective 5-year mortality rates of MPA-ILD and non-MPA-ILD patients were 44.6% and 30.7%. Patient survival was better for indeterminate for UIP and alternative diagnosis than for UIP HRCT pattern (*P* = 0.023, *P* = 0.011, respectively; overall analysis: *P* = 0.023) (Fig. 3B). The 5-year mortality rate for UIP was 61.1% (median survival: 4.1 years), that for probable UIP was 43.4% (median survival: 6.4 years), that for indeterminate for UIP was 15.2% (median survival: 7.7 years), and that for the alternative diagnosis was 0%. The patients with a honeycomb showed a worse tendency for survival than did the patients without this finding, although the difference was not significant (*P* = 0.084) (Fig. 3C). The respective 5-year mortality rates of the patients with and without a honeycomb pattern were 47.8% and 30.1%. There was no significant difference in survival curves between the patients with and without CPFE (*P* = 0.411). The patients with anterior upper lobe honeycomb-like lesion showed significantly poorer survival than those without this finding (*P* = 0.018) (Fig. 3D). Their respective 5-year mortality rates were 49.9% and 28.5%. Older age, anterior upper lobe honeycomb-like lesion on HRCT, higher C-reactive protein (CRP) level, and the development of DAH were significant predictors of mortality by univariate and multivariate Cox proportional hazards analysis (Table 4).

**Table 3:** Cause of Death in MPO-ANCA-positive ILD Patients with and without MPA

	MPA-ILD (n = 22)	Non-MPA-ILD (n = 39)
*Deaths*		
Total	10	16
Acute exacerbation of ILD	1	4
Diffuse alveolar hemorrhage	3	0
Chronic progression of ILD	1	3
Lung cancer	0	4
Respiratory tract infection	2	2
Others	3	3

*ILD* interstitial lung disease, *MPA* microscopic polyangiitis

**Table 4:** Analysis of Predictors of Mortality in the Patients

	Univariate Cox regression			Multivariate Cox regression		
	HR	95% CI	*P*-value	HR	95% CI	*P*-value
Age	1.054	1.003, 1.107	**0.039**	1.070	1.008, 1.135	**0.026**
Male	1.534	0.688, 3.420	0.296	−		
Current/ex-smoker	2.094	0.871, 5.033	0.099	−		
BMI	0.999	0.872, 1.144	0.985	−		
*HRCT pattern*				−		
UIP	1.000	Reference				
Probable UIP	0.559	0.201, 1.554	0.265			
Indeterminate for UIP	0.295	0.080, 1.083	0.066			
*CT sign*						
Emphysema	1.513	0.559, 4.092	0.415	−		
Anterior upper lobe honeycomb-like lesion	2.751	1.151, 6.575	**0.023**	3.354	1.304, 8.629	**0.012**
Increased attenuation around honeycomb or traction bronchiectasis	1.038	0.468, 2.301	0.927	−		
Honeycomb	2.175	0.881, 5.37	0.092	−		
Concomitant with MPA (MPA-ILD)	1.056	0.463, 2.408	0.897	−		
Acute exacerbation of ILD	1.329	0.524, 3.368	0.549	−		
Diffuse pulmonary hemorrhage	11.363	3.619, 35.67	** < 0.001**	9.822	2.977, 32.406	** < 0.001**
CRP	1.196	1.051, 1.363	**0.007**	1.250	1.081, 1.444	**0.003**
%FVC	0.986	0.956, 1.017	0.381	−		
%DL_CO_	0.990	0.962, 1.020	0.526	−		
Anti-inflammatory agent	0.952	0.425, 2.129	0.904	−		
Anti-fibrotic agent	0.629	0.146, 2.704	0.533	−		

BMI = body mass index; CI = confidence interval; CRP = C-reactive protein; CT = computed tomography; DL_CO_ = diffusing capacity of the lung for carbon monoxide; FVC = forced vital capacity; HR = hazard ratio; HRCT = high-resolution CT; ILD = interstitial lung disease; MPA = microscopic polyangiitis; UIP = usual interstitial pneumonia

## Discussion

In some patients, chronic ILD preceded the development of MPA by 1–10 years [17]. Using the previous HRCT classification [18, 19], the most frequent pattern of ILD with MPA is that of UIP (50–57%), followed by nonspecific interstitial pneumonia (7–31%), and desquamative interstitial pneumonia (14%) [20]. UIP is also the most common abnormal pattern in cases of MPO-ANCA-positive ILD but without generalized involvement [20]. The present study also showed these above characteristics and findings; however, we have obtained some new knowledge.

First, HRCT pattern classification (i.e., UIP, probable UIP, indeterminate for UIP, and alternative diagnosis) based on the recent IPF guideline was useful to predict prognosis in the MPO-ANCA-positive ILD patients. In the present study, the patients with indeterminate for UIP pattern accounted for a quarter of the MPO-ANCA-positive ILD patients and had a better prognosis than those with UIP pattern, although there was not a significant difference between indeterminate for UIP and probable UIP pattern. The presence of a radiological honeycomb pattern was not a significant predictor of poor prognosis in the present study. We speculate that in previous studies, if patients had a radiological honeycomb, they most probably had UIP pattern rather than other patterns [12]. Therefore, the position of indeterminate for UIP regardless of having a honeycomb is thought to be meaningful. In another view of this point for comparison with IPF, the present study showed UIP pattern (using the recent classification) to have a poor prognosis as patients with this pattern had a short median survival time (4.1 years) similar to that for IPF. However, previous studies showed the survival time in MPO-ANCA-positive patients with UIP pattern (using an older previous classification) tended to be longer than that for IPF [2, 3]. Importantly, 4–40% of the previously studied cases do not fit any specific HRCT pattern of the previous classification of IIPs [20]. Taken together, the recent IPF classification might be useful for prognostic analysis and aid in the assessment of appropriate strategies of diagnosis in MPO-ANCA-positive ILD studies.

Second, the radiological finding of increased attenuation around honeycomb or traction bronchiectasis was frequently found (39.3%) as in previous studies because MPO-ANCA-positive-ILD shows more prominent inflammatory cell infiltration, lymphoid follicles with germinal centers, and cellular bronchiolitis [2, 6, 16]. More than half of the patients with indeterminate for UIP pattern had this radiological finding in the present study. Therefore, we also thought this specific radiological finding is important in the differential diagnosis of IPF.

Third, surprisingly, anterior upper lobe honeycomb-like lesion was also very frequently (29.5%) observed in the MPO-ANCA-positive ILD patients. We recently reported that the tendency for this radiological finding is higher in patients with RA-ILD (22%) rather than SSc-ILD (8%) or polymyositis/dermatomyositis ILD (8%) [15]. This radiological finding is thought to occur due to focally destroyed lung as a result of highly inflamed airways [15]. MPO-ANCA-positive ILD and RA-ILD may resemble diseases showing a high frequency of small airway disorder [2, 6, 15]. This radiological finding might be specific not only in patients with RA-ILD but also those with MPO-ANCA-positive ILD. Other studies have shown difficulty in distinguishing MPO-ANCA-positive ILD from IPF by HRCT [2, 21]. Some patients with ILD during follow-up developed MPO-ANCA-positive conversion or MPA [3, 4]. Therefore, the presence of anterior upper lobe honeycomb-like lesion may be an indicative predictor of MPO-ANCA-positive ILD or RA-ILD in these patients and thus can be useful information for clinicians. However, this radiological finding was a poor prognostic factor as were older age and a history of DAH. Fibrotic changes such as reticular shadows, traction bronchiectasis, and honeycomb pattern are found predominantly in the lower and outer regions of the lung [5, 22]. In the present study, 61% of the patients with anterior upper lobe honeycomb-like lesion also had honeycomb in the lower lobe as a result of the progression of lung fibrosis. Considering these results, the lesion in the anterior upper lobe may develop when MPO-ANCA-positive ILD progresses to some extent, but it is not present in the initial phase of the disease. Because we could not draw a firm conclusion, further studies of the radiological course of the disease over a long period may be needed.

Fourth, non-MPA-ILD patients had higher frequency of indeterminate for UIP pattern, whereas the MPA-ILD patients had higher frequency of UIP pattern and lower frequency of indeterminate for UIP pattern. MPO-ANCA positivity was associated with subsequent MPA development in some patients [2–4]. Because repeated episodes of alveolar hemorrhage due to pulmonary capillaritis could be the pathogenesis of progressive pulmonary fibrosis, and MPO-ANCA may play a direct role in the pathogenesis of progressive pulmonary fibrosis [23–25], to sum up, we might see non-MPA-ILD (indeterminate for UIP pattern) patients experiencing a sequence of events (i.e., pre-stage) that lead to MPA-ILD (progressive stage: UIP).

Fifth, regardless of the presence of MPA, which did not have a significant influence on prognosis, the cause of death is different between the two entities. AE of ILD occupy an important place in the outcome of ILD patients without MPA [26] as shown in the present study. All forms of ILD are at risk of AE and have a similar outcome to AE-IPF [26]. Because data from studies in patients with progressive fibrotic ILD suggest that anti-fibrotic therapy could have a role in preventing AE-ILD [27], this therapy may also improve prognosis in MPO-ANCA-positive ILD, particularly in non-MPA-ILD. However, the presence of a history of DAH is associated with poor prognosis and mortality in MPA-ILD. Several studies suggested that anti-inflammatory therapy can reduce the risk of MPA-related DAH development [4]. In fact, the present study showed that none of the patients with a history of DAH received anti-inflammatory therapy at the time of DAH. As Kagiyama et al. mentioned, anti-inflammatory therapy in patients with MPO-ANCA-positive ILD might offer some benefit in reducing the development of DAH because DAH is normally progressive and fatal [3]. Moreover, Sun et al. reported poorer prognosis in MPA-ILD and non-MPA-ILD patients with elevated inflammatory markers (CRP or erythrocyte sedimentation rate) compared with non-MPA-ILD patients with normal inflammatory markers [28]. Our study also showed higher CRP to be a poor prognostic factor, although there was no difference in survival between the MPA-ILD and non-MPA-ILD patients. However, the frequency of the patients with non-MPA-ILDs receiving anti-inflammatory agents differed greatly; more than 90% as reported by Sun et al. [28] versus 35.9% in our study. Therefore, anti-inflammatory agents may improve the prognosis of non-MPA-ILD patients, particularly in those having elevated inflammatory markers. Taken together, further accumulation of studies is warranted to clarify the clinical effectiveness of anti-inflammatory and anti-fibrotic therapy for MPO-ANCA-positive patients with ILD.

Our study has several limitations. First, it is a single-institution retrospective study, which introduces referral bias and limits the ability to generalize our findings. Second, it is possible that patients with incidental MPA may have been excluded from those with non-MPA-ILD because the differential diagnosis of MPA is difficult. For example, DAH may be misdiagnosed as an AE of ILD. Third, we could not quantitatively assess the HRCT patterns, and this will be an issue for further investigation. Fourth, because the protocol for ILD diagnosis is not standardized in our hospital, clinical practice varies between different clinicians, such as pulmonologists and rheumatologists. Therefore, these data may mask potential differences.

## Conclusions

Radiological indeterminate for UIP pattern resulted in a better prognosis than UIP pattern and therefore was a useful HRCT classification in MPO-ANCA-positive patients with either MPA-ILD or non-MPA-ILD (i.e., IIPs). Increased attenuation around honeycomb and traction bronchiectasis was a specific radiological finding. In addition, anterior upper lobe honeycomb-like lesion was also found to be a highly frequent radiological finding that was a significant negative prognostic factor. Our results might help to provide more information in diagnosing patients and predicting the prognosis in MPO-ANCA-positive patients with ILD. This challenge in clinical practice will require further accumulation of patients to better achieve a prompt diagnosis so that appropriate treatment decisions can be made.

## Data Availability

The datasets used and/or analyzed during the current study are available from the corresponding author on reasonable request.

## Abbreviations

AE: Acute exacerbation
ANCA: Antineutrophil cytoplasmic antibody
BMI: Body mass index
CPI: Composite physiologic index
CRP: C-reactive protein
CPFE: Combined pulmonary fibrosis with emphysema
DAH: Diffuse alveolar hemorrhage
%DL_CO_: %Diffusing capacity of the lung for carbon monoxide
FEV_1_: Forced expiratory volume in 1 s
FVC: Forced vital capacity
HRCT: High-resolution computed tomography
IIPs: Idiopathic interstitial pneumonias
ILD: Interstitial lung disease
IPF: Idiopathic pulmonary fibrosis
KL-6: Krebs von den Lungen-6
MPA: Microscopic polyangiitis
RA: Rheumatoid arthritis
SP-D: Surfactant protein D
SSc: Systemic sclerosis
